# Philadelphia Obstetrical Society

**Published:** 1889-06

**Authors:** J. M. Baldy

**Affiliations:** Secretary


### PHILADELPHIA OBSTETRICAL SOCIETY.

THURSDAY, April 4, 1889.

THE Vice-President, Dr. W. H. H. Githeus, in the chair.

Dr. G. E. SHOEMAKER described an improvised waterproof drainage-pad for operations.

The only point of the arrangement here described is that it may be improvised in any household, even the poorest; and it is intended that it shall take the place of the excellent device so widely advertised by an instrument-maker, except in emergencies. It happens to every one, however, to be called upon to do various minor operations when out of the reach of all formal apparatus, and in a number of cases where it was desirable to use water freely without wetting the bed or the patient, the writer has obtained the greatest comfort and satisfaction by the following means:—

The necessary material, which can be had anywhere, consists of a sheet or thin coverlet and a piece of rubber cloth or table oil-cloth.

The sheet is folded twice, and then made into a tight roll about three or four feet long to form the rim. This roll, laid near the edge of the bed or table, is bent into the form of the letter O, with a six-inch opening on one side of the O, the ends of the roll at this opening being fastened by safety-pins strongly to the edge of the mattress or the cover on the table.

When the rubber cloth is thrown loosely over this, a basin is formed which is open at the edge of the bed, and fluids readily find their way into a vessel on the floor to which the rubber cloth leads. Even a prolonged perinæum operation, under constant irrigation, may be accomplished without any disarrangement or leakage. The same arrangement will be found to be of great assistance to patients in that troublesome procedure, the daily hot-water douche.

This may, of course, be called only an adaptation of the idea in the advertised pad before referred to. It may also be called a modification of the waterproof sheet which every woman has used on her bed since the deluge. The only object here is to call attention to the fact that we can, any of us, by simply making a curved ridge in this sheet, and paying some regard to the ordinary laws of hydrostatics, make for ourselves in five minutes a very great convenience, and save our patients and their attendants a good deal of trouble and annoyance.

Dr. W. S. Stewart narrated the removal of a large, adherent, degenerated, parovarian cyst:

A young lady, aged 24 years, was brought to our hospital from New Jersey, by her physician, for examination and such treatment as should be determined on. I found in the left iliac region a hard mass, which, at first examination, seemed to be solid; but a more careful examination with the finger in Douglas' cul-de-sac, with palpation from above, revealed some fluctuation. On moving the uterus, the mass was found to be adherent to that organ, causing some doubt as to whether or not it was a true ovarian trouble. In consultation with my *confrère*, Dr. Montgomery, it was decided to remove the tumor, as from the history it evidently caused much suffering, with rather increased pain, distress, and irregular menstruation, and was developing more or less rapidly.

Two days later the patient was put on the table, and she almost died from the ether before the operation began. I was kindly assisted by the resident Dr. Hughes, chief of clinic, Dr. West, and Dr. Dorman. On reaching the upper portion of the tumor, I found it adherent to the omentum, to the small bowel, to the walls of the abdomen, to the uterus, and to every part with which it was approximated. It was also deeply seated in the pelvis. On introducing the trocar, I found that the tumor was filled with pus. The liquid degenerated, and we had a pent-up septic fluid in an almost aseptic condition. I rapidly removed the disintegrated sac from the parts to which it was attached, working as rapidly as possible, for the patient seemed to be going to die every moment. Not finding any pedicle, I was obliged to dissect the sac off as carefully as possible, and was delayed some time in getting it off. Considerable oozing, but no special bleeding occurred. I found the adhesions to the uterus so firm that it was impossible to separate the sac, and if I had used a knife, considerable time would probably have been required in ligating the vessels. I therefore transfixed the side of the uterus with a ligature, and tied both above and below, and clipped off the margin as close as possible without affecting the ligature. Where the sac penetrated deeply into the tissues of the pelvis, I ligated as closely as possible and clipped off the remaining portion of the sac. The pus escaped considerably through the pelvis, and I thoroughly irrigated with pure warm water and stitched up the wound, leaving a drainage-tube in position. The patient was returned to her room with a temperature of 96°, almost moribund. Under the use of restoratives, hot bottles, hot applications, and a hot room, she soon regained her normal temperature, and made a speedy recovery without an untoward symptom. The stitches were removed on the eighth day, and the drainage-tube allowed to remain until the ninth day. The present prospects are that the patient will entirely recover, and is now (third week) going about her room in the hospital.

As bearing on the cause of this trouble, I would say that I have learned that she lived on a farm, and that four years ago she took the part of a man in the harvest-field. Her work was pitching the sheaves from a platform in the barn up into a higher portion of the mow. Not having the strength to use the long-handled fork, she put her elbow down on the affected side, and with this as a fulcrum, and the other hand as a pry, she threw the sheaves up. In this way, possibly, she injured herself. This is a suggestion worth knowing, as a possible cause for this development. At this time she was wearing a corset, and this would confine everything so that the pressure of the elbow caused an additional strain or possible contusion.

DISCUSSION.

Dr. M. PRICE.—One point that I have noticed in regard to these pus tumors is, that the danger of the operation does not seem to be increased by the fact that they are filled with pus. The patient referred to had probably been in a septic condition at the time of operation, and the moment that the tumor was removed and thorough drainage instituted, her chances probably were as good, if not better, than in a case of simple tumor not in a sloughing condition. I have never seen a tumor filled with pus give any trouble after removal. So far as I know, the patients have always done well.

Dr. J. M. BALDY.—I must disagree with Dr. Price. It seems to me that the presence of pus and of a septic condition would considerably increase the risks of operation. In the removal of a cyst in which there had been no septic trouble, and before suppuration had taken place, the woman would be in good condition, and probably have suffered from no symptoms, save, perhaps, those of slight enlargement. In a case of that kind the risks would be small. Where a woman becomes septic from whatever cause, the risks are seriously increased.

In regard to the cause suggested, I think that there is no very good basis for assuming that this had any effect. I do not think that the pitching of sheaves and the pressure of the elbow would cause the development of such a tumor. This might have been an incidental exciting cause, but that it was the primary exciting cause we have not sufficient ground for believing. Many women develop tumors without having any severe labor of that kind. On the other hand, I have seen women who have performed such labor daily, to the severity of which I can personally testify, and never develop anything like ovarian trouble. The wholesome exercise of working in the field would, in a healthy woman, predispose rather to good health than to disease.

Dr. M. PRICE.—It is a well-established fact in surgery that a recent injury in a previously healthy individual requiring a surgical operation is more dangerous than where the operation is for an old injury. The chances of the second patient would be a hundred-fold better. I had my limb broken. A healthier boy never lived, and for six weeks it was a struggle for life. A year later I had the limb amputated, and I can testify that I have suffered more from the extraction of a tooth than from that operation. If the limb had been operated on at the time of the injury, I should probably have died. I was not used to suffering. There had been no preparation. I do not pretend to say that the presence of pus gave the patient a better chance, but the suffering prepared her for a surgical procedure which, in her case, would be more successful than it would be in a case of simple tumor with adhesions. In uncomplicated ovarian tumor, the operation is one of the simplest in surgery. The case reported was probably one of intra-ligamentous cyst, or perhaps a twisted pedicle. If the operation had been performed before pus appeared, with these strong, unchanged adhesions, her chances would not have been so great as after sloughing had taken place, and degeneration of the adhesions had begun. There was less hemorrhage and less shock. The patient had been prepared for what had to be done.

Dr. W. L. TAYLOR.—I would agree with Dr. Baldy that the removal of a sloughing cyst would cause greater risk to the patient than the removal of a simple ovarian tumor. A patient with a sloughing cyst is necessarily suffering from septic trouble. She is weak and depressed, and her vital powers are lessened. In an ovarian cyst the vital forces are in a good condition for operation. This is the only point to which I would refer, as I did not hear the paper.

Dr. STEWART.—I think that both Dr. Baldy and Dr. Price may be right. Where the septic condition has not reduced the patients to such an extent as to preclude their recovery, they often resist shock and recover rapidly. The shock is less severe than in an operation in a patient in vigorous health. I can understand this, and have seen it in some cases.

In my case the patient scarcely survived the operation; but when the effects of the shock had passed off, her recovery did not seem to be influenced whatever.

Dr. J. HOFFMAN read the following:—

"Craniotomy for a Case of Hydrocephalus, with a Discussion of the Technique of the Operation, together with a Consideration of the Conditions that demand it."

At midnight, of December 23, 1888, I was called by a midwife to see a woman. She was unable to deliver, after, as I afterwards found out, continual effort for four hours. I found the woman much worn out by her pains, which were ineffectual, though her pulse and condition were, all in all, good. Examination showed a large head, well engaged, lying transversely in the pelvis, the occiput to the left.

I at once put on the Poullet forceps, but though they were accurately applied, was unabled to rotate the head, the forceps finally slipping. After a great deal of difficulty, I again succeeded in applying them, with like result, slipping on traction. A third effort to apply them was only successful after placing the woman on her side. Traction was, however, no more successful than before. I then desisted from further efforts at delivery, two hours having elapsed, and brought Dr. Joseph Price in consultation. Dr. Price, after a great deal of trouble, succeeded in applying the Tarnier traction forceps, with no better success, however, than had followed the use of the Poullet instrument. From the constant slipping of his forceps, Dr. Price suspected a hydrocephalic head, and so expressed himself. I, on the contrary, thought otherwise, as the bones while not so firm and resisting as usual did not seem to be sufficiently flaccid to indicate hydrocephalus, at least to me. Events, however, proved the correctness of Dr. Price's suspicion, or rather diagnosis; for there being no heart sounds when the head was perforated, the rush of water left no doubt as to the true condition. The instruments used were those presented to-night, as they already have been before. They consist of a crushing forceps, which, from its pelvic curve, is as readily introduced and applied as the ordinary forceps. The non-fenestrated blades afford the safety of a speculum for perforation, and leave no manipulation necessary after that part of the operation has been performed. All consideration of this subject seems to take for granted that the crushing instrument in all craniotomy procedures must be applied after perforation. This, it seems to me, supplies one of the greatest dangers of the operation, and conduces to an unnecessary fatality. The preapplication of the crushing instrument not only protects the maternal soft parts from the danger of injury by the perforator, but also a more exact adjustment, by a gradually applied force as the head is reduced and its contents evacuated by the perforator. The ease of application of this instrument can be appreciated by any one familiar with the ordinary forceps.

The point of the perforator Dr. Price has intended to be protected by the buckskin finger, and the skull pierced through it. This is, however, not really necessary, as the speculum afforded by the crushing blades, together with that afforded by the introducing finger, makes the leather unnecessary.

The combination of instruments afforded in this craniotomy set seems to leave nothing further to be desired, even if further destruction of the fœtus is necessary, than the mere reduction of the head. For the consideration of the conditions which demand this operation, there is at present, perhaps, a greater necessity than the mere statement of its technique with any set of instruments whatever. Many of our recent writers apparently desire to condemn it in all cases whatsoever upon the living fœtus without exception. As a type of these may be taken the views of Dr. Busey in the "American Journal of Obstetrics," January, 1889. These writers, of which Dr. Busey may be taken as a type, fail to appreciate the fact that we need go back no further than Hodge to find that, in cases where the short diameter of the pelvis is two inches or under, the Cæsarian operation is to be preferred, as affording a better prospect for the mother, while having the strong recommendation of affording a good prospect of safety to the child; this, too, before the improved Cæsarian operation was devised. These writers seem, too, to fail to appreciate that, long ago as the writer referred to, to go no further back, the early performance of the Cæsarian section was specifically stated as justifying strong hopes for "the salvation of both mother and child." It is not the purpose of this paper to discuss the relative merits of the Cæsarian section and craniotomy, nor the comparative values of the mother's and the infant's life. It is not possible to avoid, nevertheless, the observation that those writers who unhesitatingly apply the statistical method at arriving at conclusions relative to these in favor of the first operation, seemingly forget that the dangers of craniotomy almost entirely lie within the limits already admitted into the domain of legitimate Cæsarian section, and that outside of these cases the danger to the mother is almost absolutely nothing, as admitted by Lusk in his late discussion. They seem, too, to consider that craniotomy, to be successful, must be done by the expert, and that the Cæsarian section is the safer, no matter by whom performed. To this we submit a positive disagreement, though even Mr. Tait has gone so far as to say in effect that the removal of the pregnant uterus, is a simple operation. Dr. Busey refers to the "dream of Tyler Smith, as to the abolition of craniotomy from the obstetric practice."

When we consider the paper of Tyler Smith, to which reference is made, we can readily understand how opportune was the plea. The table of cases therein quoted from cases in "British Practice," affording excuse for craniotomy, state twenty-five indications for its performance, among which are, to-wit: arm or shoulder presentations, rupture of the uterus, face presentations, bands or cicatrices in the vagina, placenta prævia, rigidity of the perinæum, occipito-posterior presentations, etc. "With such 'indications' as these, there was need of a voice crying in the wilderness."

The point to be here considered is, whether the decrial of the abuse of any operation necessarily implies that there is never any requirement or justification of such operation. We think not. No one will dispute that where there is danger to the mother in the performance of craniotomy, the conservative operation of Cæsarian section should be performed. On the other hand, where there is no danger to the mother whatever, I consider it questionable whether any obstetrician here present would subject his own wife to the danger of a capital operation in order to save the life of the child. Secondly, in cases where such deformity as hydrocephalus or spina-bifida is discovered, I do not believe that the life of the child should be considered as compared with the mother's in the danger of the Cæsarian section, providing that the pelvic contraction be not so great as to bring craniotomy farther beyond the danger-line than the Cæsarian operation.

The application of the same principle in the case of monsters needs no discussion.

The woman recovered without a bad symptom.

DISCUSSION.

DR. STEWART.—I would say a word in regard to this case of hydrocephalus. I have had two or three such cases, and have had no difficulty in delivering after penetrating the skull and allowing the water to escape. I consider this an ingenious instrument, but I have used the old fashioned perforator, cutting both ways. After introducing the blades and separating them you have a free escape of the liquid. The skull then collapses, and there is no further difficulty. You can deliver then with any forceps. Such has been my experience.

DR. DANIEL LONGAKER.—I have not used this instrument of Dr. Price, but I can readily see that in a certain class of cases, *e.g.*, hydrocephalus, it would be excellent. I desire, however, to say, that in craniotomy, and especially in cases of marked deformity of the pelvis, I have used with the most marked satisfaction the cranioclast of Braun, and the perforator of Blot. I do not see how in ordinarily careful and skillful hands any injury can be done with this perforator. The trepan is certainly a safe instrument.

DR. J. PRICE.—I have discussed this matter on several occasions, but the remarks of Dr. Longaker invite me to say something. The application of the instruments mentioned is difficult. When closed they occupy one inch of pelvic space. Much damage is often done, and the mortality in craniotomy has been largely due to injury of the maternal soft parts by this instrument. In one case the sacrum was trephined with the instrument alluded to. Hodge long ago called attention to the use of the ordinary forceps as a compressor. This instrument is made on the same principle, and the strength is in the handles. You can crush anything with this instrument. Any one who can apply the forceps can apply this instrument in any pelvis where the forceps can be applied. It can be applied in a pelvis with a diameter of one and a half inches. I have seen it successfully applied by beginners in the case of dead children without doing any mischief. The instrument is used first as a speculum, second for fixation, and third for compression.

DR. LONGAKER.—It is only necessary to refer to my own experience with cranioclasis, and to confirm my favorable opinion of the operation. I will refer to a paper which can be found in the "American Journal of Obstetrics," I think, for December, 1884. Cases by the fifties and hundreds are reported without a fatal result. This is proof of the safety of cranioclasis, which I consider the better operation where there is a high degree of pelvic deformity.

DR. J. PRICE presented specimens with remarks—

I desire first to present two fresh specimens. One was very unique, removed day before yesterday, — a case of double pus-tubes and double ovarian abscess, with pus in the cellular tissue. The ovaries were cheesy shells, and they both ruptured in the removal. The pavilions were entirely gone. There was no hesitation on the part of those present in regard to the character of the fluid. It was pus. Much has been said in regard to the character of the fluid from this locality. If such liquid was removed from other parts of the body there would be no question in regard to its character. I open one of these tubes before you, and I trust that you will examine the fluid carefully.

This is a typical case of ovarian cyst, no larger than an egg, with no semblance of the pavilion. This contained fluid; but I do not claim that it was pus. The cyst was strongly adherent, and I had to shell it out.

Here is an enormous ovarian abscess, unquestionably due to gonorrhœa.

You will find that most of these tubes have been cut through, and a stick inserted. Most of them on removal were as large as the uterus. All of the patients were great sufferers. I am sometimes asked what becomes of the patients who refuse operation.

In five of these cases I had urged section from a few months to several years previously.

This specimen is from a woman to whom I urged operation five years ago. It is an enormous dermoid cyst, encapsuled by omentum. It looked like a hopeless case, but she made a good recovery.

Here are two small ovarian cysts, in which it would have been easy to guess at the diagnosis of extra-uterine pregnancy. Here are typical pus-tubes, and you can bear in mind that the character of the fluid in these cases was that of the tubes before you.

I have here a group of four or five small cysts, the removal of which I consider important. These patients suffered severe pain. These occurred in young women who were able to definitely locate the seat of the pain. One of these small cysts developed in a recently married woman 19 or 20 years of age. She saw me three days ago, three months after operation, and states that she has missed the last two periods.

This small tumor was removed by Dr. Muller, of Germantown. The ovary is healthy, and you see a very pretty parovarian cyst. The woman made a speedy recovery.

I have here two extra-uterine pregnancies. The placenta and clot in one are seen in the tube, and can be removed. This is unquestionably an extra-uterine pregnancy. In the other the placenta is inside. The specimen has been examined by Dr. Piersol and Dr. Meigs, and they state that it is undoubtedly extra-uterine pregnancy. This is a hydrosalpinx of the opposite side of the first case. Here you have a beautiful illustration of the existence of double disease. On one side desquamative salpingitis, hydrosalpinx and pus-tubes, and on the other side extra-uterine pregnancy. The second case was an example of double tubal pregnancy, and both tubes had ruptured. This woman lived after the uterus had been curetted twice, and iodine had been injected after the second operation. The bleeding continued, and until the abdomen became distended, it was not deemed necessary to do anything further.

I desire to say that four of these operations followed Emmet's operation for laceration of the cervix. If there exists any tubal disease, this is a dangerous procedure. Some one has remarked that Emmet has gone back on his operation. He has uttered a word of caution because of the mortality in the hands of some of his followers. Many of the cases come back to him. Where there has been tubal disease, many deaths have occurred, and many patients are invalids. I do not condemn the operation. I know it to be valuable in well-selected cases, when you can exclude the existence of tubal disease.

DISCUSSION.

Dr. M. PRICE.—Here again comes up the question of the preparation of the patient by the leakage that has been going on in the pus cases. I have seen at least fifty pus cases in the last two years. Very rarely is it that you can deliver the tube without some leakage and perhaps rupture. These cases have recovered and do better than some simple cases. Our nurses always prefer a pus case where a drainage-tube has been used. Where a patient is poisoned and dying, no one makes any claim that there is any advantage; but where inflammatory changes have been going on for a long time, there is, unquestionably, a preparation. I have never seen but one case of pus in the pelvis die. That case died from starvation from the nurse drinking the milk. These cases recover if the enucleation has been done with care, and irrigation and drainage properly performed.

DR. HOFFMAN.—In regard to the gonorrhœal origin of these troubles, I would say that two weeks ago I had a child two weeks old brought to me with sore eyes. I applied nitrate of silver, and gave explicit directions as to treatment. In three days the child lost its sight. I found that the mother had gonorrhœa of the most virulent form. I also found ovarian and tubal trouble very marked on one side. She had been married only a short time and previously had known no trouble. There seems to be a connection between the inflammation of the child's eyes, the gonorrhœal discharge from the vagina of the mother, and the trouble in the pelvis.

Dr. J. M. BALDY.—I do not care to say anything in regard to pus-tubes, because my views have been often expressed. I would again take exception to the view of the preparation of the patient by sepsis. It is true that in many surgical injuries better results are secured where the operation is done some time subsequently, than when it is done at once. This is not because of septic infection. Shock is here a great element. This brings up the old theory that it was better to allow ovarian tumors to reach a large size, in order that the peritoneum might be prepared, etc. We have long since given this up, and we shall quickly have to give up the idea that the patient is prepared for operation by being septically infected.

Dr. Hoffman has referred to the connection between inflammation of the child's eyes and gonorrhœa in the woman. Individual cases do not go for much. The fact that the child has inflamed eyes does not indicate positively the existence of gonorrhœa. The nurse's hands being contaminated by the septic lochial discharges may infect the child's eyes.

In discussing this matter in the Pathological Society the other night, the president stated that he had seen unquestionable gonorrhœal pus-tubes removed from a single woman. On inquiry, however, he admitted that the woman had had two criminal abortions a short time before. This was probably the cause of the inflammatory trouble, and this is the history of many of these cases. I protest against the view that assigns gonorrhœa as the cause of all of these cases. It is a dangerous teaching for ourselves as a profession, and it is dangerous teaching to the laity. If we teach the laity that all these cases, or most of them, are of gonorrhœal origin, we shall cause an unlimited degree of marital unhappiness throughout the country, and shall cause irreparable family troubles. We certainly have to have better and more scientific ground than mere clinical histories before we can accept this extreme view. Dr. Hoffman, in a recent discussion, cited the statistics of Bernetz and Goupil as a proof of this view. Out of ninety-nine cases, about forty-six were of gonorrhœal origin, and these were in the lowest class of women. Even by these picked statistics, and amongst this low class, over half were of septic origin, and many of the supposed gonorrhœal ones I would be inclined to dispute. I must adhere to my opinion, that, by all odds, septic infection is the most common cause.

J. M. BALDY, Secretary.

